# The Dutch Social Interaction Anxiety Scale and the Social Phobia Scale: Reliability, Validity, and Clinical Utility

**DOI:** 10.1155/2014/360193

**Published:** 2014-02-12

**Authors:** Edwin de Beurs, Deirdre Tielen, Lisa Wollmann

**Affiliations:** ^1^Department of Psychiatry, Leiden University Medical Centre, Albinusdreef 2, 2333 ZA Leiden, The Netherlands; ^2^Rivierduinen Mental Health Institution, Postbus 405, 2300 AK Leiden, The Netherlands; ^3^Department of Clinical Psychology, Leiden University, Wassenaarseweg 52, 2333 AK Leiden, The Netherlands

## Abstract

The social interaction anxiety scale (SIAS) and the social phobia scale (SPS) assess anxiety in social interactions and fear of scrutiny by others. This study examines the psychometric properties of the Dutch versions of the SIAS and SPS using data from a large group of patients with social phobia and a community-based sample. Confirmatory factor analysis revealed that the SIAS is unidimensional, whereas the SPS is comprised of three subscales. The internal consistency of the scales and subscales was good. The concurrent and discriminant validity was supported and the scales were well able to discriminate between patients and community-based respondents. Cut-off values with excellent sensitivity and specificity are presented. Of all self-report measures included, the SPS was the most sensitive for treatment effects. Normative data are provided which can be used to assess whether clinically significant change has occurred in individual patients.

## 1. Introduction

Two types of fears have been found to dominate social phobia: fears of initiating or managing social interactions and fears of being observed or being the center of attention [1]. These have been termed interaction versus performance fears [2]. The two most frequently used measurement instruments to assess both aspects are the social interaction anxiety scale (SIAS) and the social phobia scale (SPS). The SIAS is intended to assess social interaction anxiety; “distress when meeting and talking with other people” [3, page 457], whereas the SPS assesses performance anxiety; “anxiety and fear at the prospect of being observed or watched by other people, and in particular, where the individual expresses distress when undertaking certain activities in the presence of others” [3, page 457]. Both scales were developed in 1989 by Mattick and Clarke and published in 1998. Matttick and Clarke generated an initial pool of 164 items from existing anxiety measures. After removing 85 redundant items and four items on which a panel of judges could not agree on the type of social anxiety being assessed, 75 items remained. These were administered to respondents (two samples from the general population and a clinical sample of 243 patients with social phobia, 16 with simple phobia, and 13 with agoraphobia) and the resulting data were submitted to statistical analyses. These resulted in a final set of 39 items: 19 in the SIAS and 20 in the SPS. Eventually, one item was added to the SIAS (“I find it easy to make friends of my own age”) resulting in two measures with 20 items each. The benefits of the scales are that they are concise and provide separate scores for performance and interaction anxiety [4].

The scales have good psychometric properties as attested by satisfactory reliability indices: internal consistency, determined by Cronbach's *α*, ranged from 0.88 to 0.93 for the SIAS and 0.89 to 0.94 for the SPS [3, 5]. Test-retest reliability was *r* = 0.92 for the SIAS and ranged from 0.91 to 0.93 for the SPS (for 4- and 12-week intervals, resp.). Validity is also supported by research. Convergent and discriminant validity was reinforced by findings of Heimberg et al. [5] showing higher associations for the SPS than the SIAS with other measures of performance anxiety, such as the performance subscale of the Liebowitz social anxiety scale (LSAS; [1]). Conversely, the SIAS was more strongly associated with the social interaction subscale of the LSAS. Criterion-related validity of both scales was demonstrated by Heimberg and colleagues who found significant differences between social phobia patients and community-based respondents [5]. Further support for criterion-related validity is found by the fact that the scales were able to differentiate between anxiety disorder patients (e.g., social phobia patients and agoraphobics) [3]. According to a more comprehensive study by Brown et al. [6] which compared SIAS and SPS scores of patients with various anxiety disorders, the SIAS distinguished social phobia from all other anxiety disorders, but the SPS did not discriminate between social phobia patients and patients suffering from panic disorder and agoraphobia.

While the SIAS and the SPS may themselves encompass subscales, results from different research groups employing (confirmatory) factor analyses have yielded different factor structures. Mattick and Clarke [3] carried out exploratory factor analyses on the SIAS and SPS. The SIAS appeared to consist of a single factor, but within the SPS they found three subscales: (a) scrutiny of being observed in a variety of places, (b) specific fears of performing certain behaviors in public, such as writing or drinking, and (c) fears of being viewed as sick, odd, or losing control in front of others. Given their development from a single item pool, the SIAS and SPS can be considered subscales of a single larger measure. Following this logic, Safren et al. [7] undertook a joint analysis of the 40 SIAS and SPS items with confirmatory factor analysis. They failed to find adequate fit for a two-factor model. Three factors were found with a subsequent exploratory factor analysis: (a) interaction anxiety, measured by 17 of the 20 SIAS items, (b) anxiety about being observed by others assessed by 11 SPS items, and (c) fear that others may notice symptoms of anxiety, measured by nine SPS and three SIAS items. Whether or not reliable and valid subscales can be discerned is important for the construct validity of the SIAS and SPS, but it also bears relevance for the clinical utility of the instrument. More detailed information on what situations a given patient predominantly fears will allow for individualized treatment planning [6]. The SIAS and the SPS render valuable information for this purpose.

Translations of the SIAS and SPS in German [8] and Spanish [9] are available and have been evaluated on their psychometric properties. These properties appeared satisfactory, with good reliability and validity indices. We undertook the task of developing a Dutch version of both scales. The original scales were translated according to the recommendations by van Widenfelt and colleagues [10] and we will report on the psychometric properties of the translated measurement instrument. First, item characteristics were investigated (mean, SD, skewedness, and kurtosis). Next, construct validity was investigated with confirmatory factor analysis. The fit of a single factor and various multifactor models were evaluated separately for the SPS and SIAS and jointly for the 40 items. The reliability coefficients (internal consistency) were calculated and various aspects of the validity of the subscales were evaluated. Convergent and discriminant validity was investigated by calculating correlation coefficients between subscales of the SIAS and SPS and other self-report instruments measuring similar and dissimilar concepts. Criterion-related validity was assessed by comparing scores of patients meeting DSM-IV criteria for social phobia and community-based respondents. The sensitivity and specificity of the SIAS and SPS to detect social phobia were established with ROC analysis. Finally, we investigated the sensitivity of the scales for change over the course of treatment as compared to other outcome measures.

## 2. Method

### 2.1. Subjects

The SIAS and SPS, together with a number of other measures, were administered to a consecutive sample of patients seeking treatment at outpatient clinics of Rivierduinen, a large mental health clinic serving the population of Leiden and surrounding communities. All patients (*N* = 359) met the criteria for DSM-IV social phobia, and 287 (80%) were diagnosed with generalized social phobia. The mean age of the sample was M = 33.4, SD = 11.0, range of 18–60.

As part of the standard intake procedure, patients completed a battery of assessment instruments to measure psychopathology. The diagnosis was assessed with a standardized diagnostic interview, the mini-international neuropsychiatric interview (MINI-plus; [11]), carried out by extensively trained staff (a psychiatric nurse or a psychologist). In the assessment session, self-report questionnaires were administered through a computer program specially devised for this task and the research nurse completed several rating scales. The entire assessment session took about 120 minutes. Patients participated in routine outcome monitoring (ROM; [12]). This involved readministration of the battery of assessment instruments during their treatment in three- to four-month intervals. Participation of all patients was voluntary. Informed consent is not mandatory under Dutch law when the administration of the battery of measurement instruments (a) is part of the routinely performed intake procedure and (b) does not involve an additional risk or burden, and (c) data are analyzed anonymously. All these requirements were met in the current study.

In addition, a sample of 371 respondents from the general population was obtained by randomly approaching potential respondents on the street or in public places and inviting them to complete two short questionnaires for scientific research aiming to “investigate questionnaires for the assessment of emotional functioning.” Special care was taken to ensure that the sample was similar to the general population on relevant variables such as age, gender, and size of the place of residence (two-staged proportioned stratified sampling; [13]). 502 people were approached, of which 398 (79%) agreed to participate. A total of 371 respondents in the designated age range (18+) rendered usable data (93% of all questionnaires that had been completed and 74% of all contacted potential respondents). We compared demographic characteristics of the sample (gender, age, marital status, educational level, and religious affiliation) with the general population. This indicated that there was no sample bias. 54% of respondents (*n* = 200) were female; the mean age was M = 39.0, SD = 15.9 (range = 18–86); 69.0% were married; 52.0% were employed; 25.5% were stay-at-home wives.  Table 1 presents some additional demographic data from both samples.

### 2.2. Measures

#### 2.2.1. SIAS and SPS

The SIAS and SPS are both made up of 20 self-assessment statements each to be rated on a five-point scale, where 0 indicates an extreme level of disagreement and 5 agreement. For example, when given the statement “I feel I may blush when I am with others,” the respondent would tick 5 indicating that the statement is completely characteristic of them and 0 to state that it is unlike them.

Results are calculated by reversing the scoring of three positively worded items on the SIAS (items 5, 9, and 11) and summing up the scores. Thus, for both scales, the scores may range from 0 to 80, with higher scores indicating more discomfort or anxiety.

#### 2.2.2. Other Instruments

All patients completed the inventory of interpersonal situations (ISS; [14]) and the brief symptom inventory (BSI; [15]). The IIS measures frequency of social activities and anxiety while engaging in these activities. Both aspects are broken down into five subscales (criticizing; taking a clear position on matters; complimenting someone else; initiating a social interaction; and valuing oneself). The BSI assesses various forms of psychopathology. On this checklist of 53 symptoms, the respondent indicates to what extend they have been bothered by each symptom in the past week (0 = “not at all,” 4 = “extremely”). The BSI includes subscales for somatic complaints, depression, anxiety, phobic avoidance, and interpersonal sensitivity. The total score on the BSI is generally perceived as a highly reliable index of general psychopathology.

In addition, patient's social phobia was rated by the research nurse on the Liebowitz social anxiety scale [1]. This scale contains 24 items: 11 items describe social interaction situations and 13 describe performance situations. Items are rated twice: once for fear of social situations and again for avoidance of them. Thus, the LSAS yields four subscale scores and, additionally, the total fear and total avoidance scores. Items are rated on a four-point scale (0 = “none,” 3 = “severe”). Research nurses were extensively trained in administrating the rating scales and conducting the diagnostic interview. Each new research nurse followed intensive two-week training with an experienced nurse, before doing ratings independently. In addition, biweekly two-hour training sessions were organized throughout the study in which guest speakers taught about psychiatric disorders and videotaped patients were jointly rated by the group of research nurses to improve interrater reliability.

Diagnostic status of patients was assessed with the MINI-plus [11]. The MINI-plus is a standardized diagnostic interview comprised of 23 modules in which the DSM criteria for the main psychiatric disorders (mood, anxiety, psychotic, somatoform, and eating disorders) are investigated (DSM-IV Axis I disorders). Each module starts with one or two screening questions. If these are answered affirmatively, additional questions from the module are asked. Lecrubier and colleagues [16] report sufficient reliability for most modules. Interrater reliability ranged from *κ* = 0.88 to 1.00; test-retest reliability ranged from *κ* = 0.76 to *κ* = 0.93; validity was demonstrated by sufficient concordance with the CIDI (kappa's ranged from *κ* = 0.36 for generalized anxiety disorder to *κ* = 0.82 for alcohol dependence). For the social phobia module *κ* = 0.54. The MINI-plus identifies two subtypes of social phobia: generalized social phobia and social phobia with specific fears.

### 2.3. Statistical Analysis

**Figure 1:**
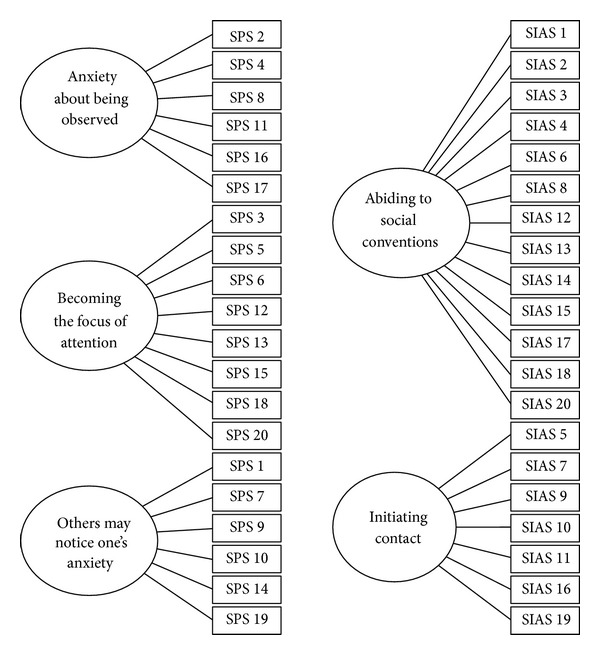
Proposed 5-factor structure for the SIAS and SPS items.

The frequency distributions of scores on the translated items were investigated (M, SD, skewedness, and kurtosis). Various factor models for the SIAS and SPS were then investigated with confirmatory factor analysis [17]. The research literature suggests that the SIAS may consist of two factors: anxiety about initiating a social interaction (“I am tense mixing in a group”) and concerns about an ongoing social interaction (“I feel I'll say something embarrassing when talking”). In the SPS, three factors are discerned: (1) anxiety about being observed by others (“I would get tense if I had to sit facing other people on a bus or a train.”), (2) becoming the focus of attention (“I feel self-conscious if I have to enter a room where others are already seated.”), and (3) fear that others will notice anxiety symptoms (“I worry about shaking or trembling when I'm watched by other people.”). Item allocation for the two models and tentative labels of the factors are depicted in Figure 1.

Reliability was investigated by analyzing the internal consistency of scales using Cronbach's *α*. Convergent and discriminant validity was evaluated by correlating the SIAS and SPS with parallel tests (bivariate correlations). Criterion-related validity was investigated by assessing the ability of the SIAS and SPS to differentiate between the patient sample and the population sample, as well as between diagnostic subgroups within the patient sample with *t*-tests. Finally, we compared the sensitivity for treatment effect of the SIAS, SPS and other instruments by testing pre-posttest differences with *t*-tests and expressing the pre- to posttest gain in an effect size index, Cohen's *d*-accent for repeated measures [(1/the pre-postcorrelation) ∗ (pre-postdifference divided by the pooled Sd)].

## 3. Results

### 3.1. Basic Psychometrics and Construct Validity (Factor Structure)

Inspection of the frequency distributions of the individual items did not reveal substantial deviation from the normal curve, implying no need to alter phrasing of any items. Some items' scores from the control sample were skewed, but this is understandable given the low prevalence of pathological social fear in the general population.

To investigate the factor structure of the SIAS and SPS, various factor models were tested on their fit with LISREL 8.30 [18]. For the SIAS, a first order single-factor model, a first order two-factor model, and a second order two-factor model (two factors grouped under a single higher order factor) were tested. Similarly, a first order single-factor model, a first order three-factor model, and a hierarchical three-factor model were compared for the SPS. Finally, the 40 items of the SIAS and the SPS were pooled and first order two- and four-factor models were compared with second order two- and four-factor models.

Fit indices for the various models are presented in Table 2. All models in the SIAS have an equally modest fit, rendering a unidimensional model as the most parsimonious solution. A first order three-factor model had optimum fit with the SPS. A confirmatory factor analysis of the pooled items from the SIAS and SPS revealed best fit for a first order four-factor model with all SIAS items on a single factor and the SPS factor allocated to 3 factors as depicted in Figure 1. This result converges with the findings on separate analyses of the SIAS and the SPS. Thus, we also analyzed the data considering the SIAS as unidimensional and the SPS as comprised of three subscales.

### 3.2. Reliability of the Scales

The reliability indices of the scales (internal consistency) and intercorrelations among items of the scales (and their range) are presented in Table 3. Reliability of the scales ranges from good to excellent: all *α* ≥ 0.80. inter-item correlations indicate sufficient concordance without suggesting the presence of redundant items. The correlation between the subscales of the SPS was substantial (intercorrelations SPS: *R*_SPS1-SPS2_ = 0.73; *R*_SPS1–SPS3_ = 0.63; *R*_SPS2-SPS3_ = 0.64), indicating a shared variance ranging from 40% to 53%, which raises some doubt on the distinctiveness of these scales.

### 3.3. Convergent and Discriminant Validity

Table 4 presents the correlation coefficients of the SIAS and SPS scales with other measures of psychopathology. Not surprisingly, the total score of the ISS scale for social anxiety has the highest correlation with the SIAS and SPS (sub)scales. Among subscales of the ISS, the strongest association is found between the SIAS score and the initiating contact score of the ISS (*r* = 0.68). Among the SPS subscales, becoming the focus of attention (SPS-2) has the highest correlation with initiating contact (*r* = 0.58). All these findings support the convergent validity of the SIAS and SPS scales. On inspection of the correlation coefficients with a more remote measure such as the BSI, we again notice high correlations between the SIAS and the interpersonal sensitivity subscale and between the SPS and the phobic anxiety subscale (and the total score) of the BSI. Discriminant validity is supported by the low correlation between the SIAS and SPS and hostility and somatic complaints. Finally, the correlation with the LSAS (a measure assessing a similar construct through a different method) is substantial. The relatively high correlation with the psychoticism subscale in the BSI (*r* = 0.55) is noteworthy. This is supposedly an indication of discriminant validity. This BSI scale is composed of items measuring “hermit-like” behavior (preferring solitude, loneliness) which might explain its high concurrence with instruments measuring social anxiety.

### 3.4. Criterion-Related Validity

The initial test for criterion-related validity of an instrument is its ability to discriminate between patients and the normal population. We compared scores on the SIAS and SPS for both groups with *t*-tests. The means, SDs, results of the *t*-tests, and the effect size of the difference (Cohen's *d*) are listed in Table 5. All scales discriminate well between patients and normal controls and statistical significance is upheld after Bonferronni corrections for multiple testing. The difference between groups is substantial whereas differences among the various subscales in criterion-related validity are small: effect size indices (Cohen's *d*) suggest that the SIAS is best able to distinguish between both groups.

We calculated receiver operating characteristic (ROC) curves for the SIAS and SPS total scores and established various cut-off values on the SIAS and SPS total score of the sensitivity and specificity. As we found a significant gender difference (SIAS: *t*(713) = 2.95; *P* = 0.003 (two-tailed), *d* = 0.22; SPS: *t*(713) = 2.89; *P* = 0.004 (two-tailed), *d* = 0.22), separate analyses were done for men and women. Both scales appear very suitable for case finding (all AUC ≥ 95). Utilizing a cut-off score for males of ≥18 on the SPS would result in only 4% of false positives and 18% true cases being missed. Setting the cut-off to ≥10 would lead to missing 4% of cases and misdiagnosing 21% as false positives (see Table 6).

Demonstrating that the SIAS and SPS scales are able to distinguish patients from the general population may be useful for certain research goals (e.g., detecting social phobics in the general population), but a test for discriminant validity of a scale should also encompass demonstration of its ability to discriminate between groups of patients. We included no patients with disorders other than social phobia in our patient sample. However, it was assessed whether the patient suffered from *generalized* social phobia or not. We compared generalized and nongeneralized social phobia on the SIAS and SPS. The mean score of patients with generalized social phobia was about 10 scale points higher (M = 37.0,  SD = 16.6 versus M = 27.4,  SD = 16.6 on the SPS, for generalized versus nongeneralized social phobia, resp.; *t*(355) = 4.02,  *P* < 0.001,  *d* = 0.61).

Finally, it is relevant to compare the mean scores of our patients on the SIAS and SPS with other studies on social phobia patients. We inspected the means of American patients by Brown et al. [6] and means of two German samples: one from a study by Stangier et al. [8] and one from Heinrichs et al. [19]. On the SIAS, Brown et al. [6] report a mean of M = 50.7,  SD = 17.0, whereas Heinrichs et al. [19] found M = 44.5,  SD = 17.4 and Stangier et al. [8] found M = 40.6,  SD = 16.6. On the SPS, the means for the three studies are M = 36.9,  SD = 17.5,  M = 36.6,  SD = 16.4, and M = 28.6,  SD = 16.2, respectively. Thus, the means on the SIAS and SPS of Dutch patients bear a closer resemblance to German than American patients. Apparently, continental patients score somewhat lower on both scales.

### 3.5. Sensitivity for Change

The SIAS and SPS were compared with various other scales regarding their sensitivity for change. Routine outcome monitoring (ROM; [12]) implied that data were collected at regular intervals of four to five months. Dependently on the duration of the treatment (or the compliance of patients with the ROM regime) assessment trajectories of different lengths were available: for some patients, only two assessments were available whereas others were reassessed four or five times, spanning a treatment period of two years. To investigate sensitivity for change, we compared the first and last available assessments (the mean posttest interval in weeks was M = 39.9,  SD = 26.8). As the same instruments were administered at pre- and posttest, a direct comparison of their ability to demonstrate change is feasible.  Table 7 presents the means (and standard deviations) of the pre- and the posttest scores and effect size indices for a relevant selection of measures.

The largest difference between pre- and posttest is found with the LSAS-anxiety rating. Among the self-report inventories, the SPS is best suited to detect change in symptoms over time. On average, the score declines by 12.0 scale points on the SPS and 11.9 scale points on the SIAS, which amounts to almost one standard deviation from pre- to post-test. This change is somewhat smaller than what was found in controlled trials. For instance, Mattick et al. [20] report for their most effective treatment condition at pretest M = 41.9; SD = 6.2 and at posttest M = 24.0, SD = 12.4, implying a reduction of almost 18 points on the SPS total score. On the total score of the SIAS, the change observed in their study was 14.2 points.

### 3.6. Reliable Change Index and Clinically Significant Change

Jacobson and Revenstorf [21] suggested criteria to determine whether change over time as assessed by an outcome measure is clinically meaningful. They proposed to considerer a change statistically reliable if it falls outside a range determined by the standard measurement error of the instrument. The measurement error should be based on the test-retest reliability of the instruments, but, when such data are unavailable, Cronbach's *α* can be used to determine this range. Jacobson and Revenstorf's formulas suggest that the SIAS' cut-off point is 13. Thus, a change of 13 scale points or more implies that statistically reliable improvement (or deterioration) has occurred. The value for the SPS is also 13 scale points. To determine whether a respondent is pathological or healthy, a cut-off point for the posttest score was proposed. This was defined as halfway between the mean of the pathological population and the normal population (taking into consideration the variance in scores of both populations). Since women tend to score higher on self-report measures for social phobia, distinct cut-off points were calculated for men and women. For men, the cut-off values on the SIAS and SPS are ≥23 and ≥15, respectively, for women ≥27 and ≥18. These cut-off values are rather stringent and considerably lower compared to what is recommended for the original English scales. Brown et al. [6] found that a score of one SD above the mean of community-based subjects (≥34 on the SIAS and ≥24 on the SPS; [5]) discriminated best between patients and controls.

When the criterion for reliable change and the cut-off value are combined, there are five possible outcomes: recovery (reliable change and a transgression from pathological to healthy), improvement (merely reliable change), stable, deterioration, and relapse (reliable change and a transgression from healthy to pathological). When applied to the SPS, pre-posttest scores of our clinical sample 28.4% could be considered recovered; 18.3% improved; 49.1% remained unchanged; 4.1% deteriorated; no patients changed from healthy to pathological. This result again illustrates the stringency of the criteria, leading to a conservative estimate of treatment outcome in clinical terms.

## 4. Discussion

Strengths of the present study include that data were collected in a large sample of patients with social phobia and an equally large community-based sample (which compares favorably to studies with undergraduates as respondents). Moreover, the social phobia sample did not stem from a group selected for a randomized clinical trial but was a clinically representative sample of all patients that applied for outpatient treatment in a large mental health institution. The SIAS and SPS were evaluated on a comprehensive set of indices for reliability and validity. The results present a favorable picture of the psychometric qualities of the Dutch version of the scales.

Regarding construct validity, in particular whether the instruments are multifactorial, a number of studies have been published [3, 5, 7, 22]. The most comprehensive study to date on the factor structure of the SIAS is by Rodebaugh et al. [22] involving 445 patients with social phobia and 1689 undergraduate controls. They report a unifactorial model for the SIAS as having the best fit (when three reverse-scored items, 5, 9, and 11 are removed). Also, in the study by Heinrichs et al. [19] on the German translation of the SIAS and SPS, the SIAS appeared to be unifactorial. Regarding the SPS, we found the best fit for a 3-factor model. Other researchers also report that the structure of the SPS is multifactorial. Mattick and Clarke [3] proposed a 3-factor structure which also had the best fit in our study. Safren et al. [7] report a three-factor solution for the pooled items of the SIAS and SPS, comprised of the following: interaction anxiety (assessed 17 SIAS items), anxiety about being observed by others (measured by 11 SPS items), and fear that others will notice anxiety symptoms (assessed by 9 SPS and 3 SIAS items). In the solution by Safren et al. [7], the two factors composed of predominantly of SPS items are quite similar to our SPS solution. Anxiety about being observed and becoming the focus of attention are not distinguished, thus forming a single factor and only a few items are allocated to other factors. Heinrichs et al. [19] found five factors with exploratory factor analysis for the SPS. Three of these overlap with the 3-factor solution of Mattick and Clarke [3] and the solution put forth in the present study. We found that the correlation among the three factors of the SPS was substantial, which raises some doubt on their distinctive value. However, for clinical utilization, distinguishing fear of becoming the focus of attention from fear of being observed and that others may notice anxiety seems useful as distinct fears may be associated with distinct forms of avoidance behavior.

Of all outcome measures considered, the LSAS showed the largest change from pre- to posttest. It has been known that observer-rated scales tend to show larger improvements in patients (cf., [23]). This may in part be due to observer bias: a tendency to see a greater benefit from treatment than patients report themselves. Among the group of self-report measures evaluated in this study, the SPS proved to be the most sensitive to change but was closely followed by the SIAS. Similarly, Mattick et al. [20] report the largest change on the SPS in their controlled study. In their sample, the extent of observed change was larger than in ours. Finally, the sensitivity of the SIAS and SPS expressed in standardized units is comparable to other frequently used measures, such as the social phobia and anxiety inventory (SPAI) as Taylor and colleagues [24] report effect sizes of 1.64 and 1.75 at posttest and follow up for the SPAI.

It is noteworthy that the interpersonal sensitivity subscale of the brief symptom inventory (with only four items) also performs well as an outcome measure. For outcome assessment in routine clinical practice administration of the BSI may well suffice. Still, the total scores on the SIAS and SPS are best suited to assess the overall severity of the symptomatology at pre- and posttest and the scales are also useful for screening purposes. The SIAS and the subscales of the SPS yield four interrelated scores. This can be used to assess which aspect of social anxiety is the main concern for a given patient. This may prove helpful as it creates the opportunity of tailoring treatment to patients' individual needs.

## Figures and Tables

**Table 1:** Demographic and diagnostic characteristics of the samples (clinical *N* = 361; population *N* = 354).

	Clinical		Population	
	*N*	%	*N*	%
Gender				
Men	164	44.6	164	46.3
Women	200	55.4	190	53.7
Age				
18–25	117	32.4	93	26.3
26–45	180	49.9	144	40.7
>45	64	17.7	117	33.0
Diagnosis				
Generalized social phobia	287	79.9		
Comorbidity				
Only social phobia	109			
Comorbid mood disorder	97			
Comorbid mood and anxiety disorders	66			
Comorbid anxiety disorder	47			
Comorbid somatoform disorder	10			
Comorbid mood, anxiety, and/or somatoform disorders	30			

**Table 2:** Fit indices for competing models for separate analyses of the SIAS and SPS and a conjoint analysis of social anxiety symptoms.

	χ^2^	RMSEA	ECVI	GFI	AGFI
SIAS					
First order single factor model	596.24	0.083	1.76	0.86	0.83
First order two factor model	514.79	0.071	1.45	0.89	0.86
Second order two factor model	510.17	0.071	1.44	0.89	0.86
SPS					
First order single factor model	596.52	0.093	2.15	0.84	0.80
First order three factor model	341.65	0.061	1.20	0.91	0.88
Second order three factor model	357.61	0.058	1.26	0.91	0.88
SIAS and SPS					
First order two factor model	1902.87	0.077	6.59	0.76	0.73
First order five factor model	1570.97	0.061	5.03	0.81	0.79
Second order two factor model	1945.96	0.076	6.74	0.76	0.73
Second order five factor model	1626.33	0.061	5.22	0.81	0.79

**Table 3:** Number of items and the reliability of (subscales of) the SIAS and SPS (Cronbach's alpha, interitemcorrelations and their range). *N* = 359.

	Number of items	Alpha	*R*_ii_	*R*_ii_ range
SIAS-Total	20	0.91	0.35	−0.01–0.71
SPS-Total	20	0.93	0.38	0.15–0.71
SPS-1	6	0.87	0.53	0.33–0.71
SPS-2	8	0.85	0.43	0.29–0.59
SPS-3	6	0.80	0.40	0.17–0.56

Note: SIAS-Total: total score on the SIAS; SPS-Total: total score on the SPS.

SPS-1: anxiety about being observed; SPS-2: becoming the focus of attention; SPS-3: fear that others will notice anxiety.

**Table 4:** Convergent and discriminant validity: correlation coefficients* of the SIAS and SPS with the IIS (*n* = 140**); the BSI, and the LSAS (both *N* = 359).

	SIAS-total	SPS-total	SPS-1	SPS-2	SPS-3
ISS Anxiety					
Criticizing	0.54	0.37	0.33	0.44	0.16
Stating one's opinion	0.55	0.46	0.36	0.52	0.32
Complimenting someone	0.43	0.34	0.29	0.32	0.27
Initiating contact	0.68	0.53	0.56	0.58	0.22*
Valuing oneself	0.49	0.40	0.39	0.47	0.17*
Total score anxiety	0.69	0.54	0.49	0.60	0.30
ISS frequency					
Criticizing	0.44	0.33	0.32	0.35	0.18*
Stating one's opinion	0.42	0.37	0.30	0.32	0.37
Complimenting someone	0.25	0.01 (ns)	0.03 (ns)	−0.01 (ns)	0.00 (ns)
Initiating contact	0.53	0.44	0.47	0.41	0.27
Valuing oneself	0.38	0.37	0.36	0.38	0.19*
Total score frequency	0.56	0.46	0.43	0.43	0.34
BSI					
Somatic complaints	0.34	0.52	0.44	0.40	0.57
Cognitive functions	0.50	0.52	0.43	0.49	0.46
Interpersonal sensitivity	0.67	0.59	0.56	0.59	0.41
Depression	0.52	0.51	0.46	0.49	0.38
Anxiety	0.37	0.51	0.41	0.42	0.54
Hostility	0.38	0.40	0.34	0.36	0.36
Phobic anxiety	0.53	0.67	0.66	0.55	0.59
Paranoid ideation	0.50	0.49	0.43	0.43	0.44
Psychoticism	0.55	0.54	0.49	0.49	0.44
Total score BSI	0.60	0.67	0.59	0.59	0.59
LSAS anxiety					
Performance	0.62	0.71	0.59	0.68	0.60
Interaction	0.65	0.57	0.52	0.57	0.39
Total score anxiety	0.69	0.69	0.60	0.67	0.54
LSAS frequency					
Performance	0.50	0.61	0.54	0.55	0.52
Interaction	0.57	0.49	0.47	0.47	0.35
Total score frequency	0.58	0.60	0.55	0.55	0.47

Note: SIAS-total: total score on the SIAS; SPS-total: total score on the SPS.

SPS-1: anxiety about being observed; SPS-2: becoming the focus of attention; SPS-3: fear that others will notice anxiety; IIS: inventory of interpersonal situations. BSI: brief symptom inventory, LSAS: Liebowitz social anxiety scale.

*All correlations significant at the 0.01 level (2-tailed), except those marked with *(*P* < 0.05, two-tailed test or ns: not significant).

**The ISS was administered in routine outcome monitoring only for a limited time period.

**Table 5:** Comparison of scores of patients with social phobia and respondents from the normal population on the SIAS and SPS.

	Social phobia (*N* = 361)		Normal population (*N* = 354)		*T* (713)	d′
	M	SD	M	SD		
SIAS-total	43.7	15.4	13.7	8.6	32.1*	2.41
SPS-total	35.0	16.8	7.7	7.4	28.1*	2.10
SPS-1	12.6	6.2	3.8	2.8	28.1*	1.83
SPS-2	16.7	7.1	5.4	3.6	29.0*	2.01
SPS-3	14.5	5.6	4.4	3.2	32.1*	2.21

Note: SIAS-total: total score on the SIAS; SPS-total: total score on the SPS; SPS-1: anxiety about being observed; SPS-2: becoming the focus of attention; SPS-3: fear that others will notice anxiety.

*P < 0.001 two-tailed test.

**Table 6:** Sensitivity and specificity for finding cases among men and women at various cut-off values on the SIAS and SPS total score.

	AUC	Optimum sensitivity						Optimum specificity		
		Cut-off	Sens.	Spec.	Cut-off	Sens.	Spec.	Cut-off	Sens.	Spec.
Males										
SIAS	0.95	16/17	0.93	0.80	21/22	0.88	0.90	26/27	0.82	0.95
SPS	0.96	9/10	0.96	0.79	12/13	0.89	0.90	17/18	0.82	0.96
Females										
SIAS	0.96	19/20	0.95	0.80	25/26	0.90	0.90	32/33	0.81	0.95
SPS	0.95	12/13	0.95	0.80	16/17	0.90	0.90	21/22	0.82	0.95

Note: SIAS: total score on the SIAS; SPS: total score on the SPS.

**Table 7:** Results of comparing the mean pretest score and mean last available score and effect size (Cohen's *d*′) of the difference according to various measures (*N* = 170).

	Pretest		Last test		*T* (169)	Cohen's *d*′
	M	SD	M	SD		
SIAS	43.8	15.2	31.9	15.9	11.44*	1.23
SPS	35.0	15.8	23.0	17.1	9.95*	1.35
LSAS anxiety (*n* = 149)	38.3	12.2	27.9	15.2	9.54*	1.40
LSAS frequency (*n* = 149)	32.7	13.3	22.7	14.9	9.02*	1.31
BSI_interpersonal anxiety	2.09	0.99	1.39	1.10	8.92*	1.26
BSI_total score	1.36	0.68	0.93	0.77	8.58*	1.00

Note: SIAS: total score on the SIAS; SPS: total score on the SPS; LSAS: Liebowitz social anxiety scale; BSI: brief symptom inventory.

*P < 0.001, two-tailed test.
